# Serial Non-Invasive Measurements of Dermal Carotenoid Concentrations in Dairy Cows following Recovery from Abomasal Displacement

**DOI:** 10.1371/journal.pone.0047706

**Published:** 2012-10-31

**Authors:** Julia Klein, Maxim E. Darvin, Kerstin E. Müller, Juergen Lademann

**Affiliations:** 1 Clinic for Ruminants and Swine, Faculty of Veterinary Medicine, Freie Universität Berlin, Berlin, Germany; 2 Center of Experimental and Applied Cutaneous Physiology, Department of Dermatology, Venerology and Allergology, Charité – Universitätsmedizin Berlin, Berlin, Germany; The Scripps Research Institute Scripps Florida, United States of America

## Abstract

Maintaining the health of farm animals forms the basis for a sustainable and profitable production of food from animal origin. Recently, the effects of carotenoids on the oxidative status as well as on reproductive and immune functions in cattle have been demonstrated. The present study aimed at investigating dermal carotenoid levels in cattle recovering from abomasal displacement. For this purpose, serial *in vivo* measurements were undertaken using a miniaturized scanner system that relies on reflection spectroscopy (Opsolution GmbH, Kassel, Germany). In a first trial, repeated measurements of dermal carotenoid concentrations were performed on the udder skin of healthy non-lactating cattle (n = 6) for one month in weekly intervals. In a second trial, *in vivo* dermal carotenoid concentrations were determined in intervals in 23 cows following surgical treatment of abomasal displacement. The results show that dermal carotenoid concentrations, determined on a weekly basis over a period of one month, showed variations of up to 18% in the healthy individuals kept under constant conditions with respect to housing and nutrition. Repeated measurements during the recovery period following surgical treatment of abomasal displacement resulted in an increase in dermal carotenoid concentrations in 18 of 20 animals with a favourable outcome when compared with results obtained within 12 hours following surgery. The mean increase in dermal carotenoid concentrations in subsequent measurements was 53±44%, whereas levels decreased (mean 31±27%) in cattle with a fatal outcome.

These results indicate potential applications for reflection spectroscopy for non-invasive early detection of changes in the dermal carotenoid concentrations as a reflection of the antioxidant status in an animal.

## Introduction

As herd sizes increase, the early detection of metabolic diseases is becoming more and more important. A suitable method for the surveillance of the health status in food producing animals should be simple to use, inexpensive and reliable, and should allow the detection of diseases or deficiencies at an early stage.

Recent studies in humans demonstrate that various kinds of stress, including an unhealthy lifestyle as well as metabolic disorders and tumorgenesis, have an effect on the oxidative status [1], [2], [3], [4], [5], [6]. The latter disorders have been demonstrated to result in the generation of free radicals that cause the destruction of antioxidants. Carotenoids take part of the chain of antioxidants in the body. To this end a decrease in the carotenoid levels in blood and various tissues is regarded an indicator for antioxidant reduction [7], [8], [9], [10]. Recent investigations have shown that dermal carotenoids could serve as marker substances for the entire antioxidant status of human skin [11], [12].

Until recently, the determination of the oxidative status in humans and animals demanded the sampling of blood or biopsy materials [13], [14]. Innovative technologies such as reflection spectroscopy have been applied to determine dermal carotenoid concentrations in order to evaluate the effects of stress conditions on the oxidative status of humans. A hand-held miniaturized spectroscopic system (Opsolution GmbH, Kassel, Germany) is available, which allows serial non-invasive measurements of the dermal carotenoid concentrations in real-time on humans or animals [15], [16].

In the present study, dermal carotenoid concentrations were determined once weekly over a period of one month on six healthy cattle that were kept under the same environmental conditions and received the same diet. In addition, repeated measurements of dermal carotenoid concentrations were performed on 23 dairy cows during the recovery period following surgical treatment of abomasal displacement. The decision for abomasal displacement was taken considering the importance of this disease on the one hand as well as the absence of the influence on metabolism of carotenoids in the body, the relatively short recovery phase and the good comparability of the animals after surgery on the other.

## Materials and Methods

### Miniaturized spectroscopic system (MSS)

A LED-based compact scanner system (Opsolution GmbH, Kassel, Germany) was used for non-invasive determination of the dermal carotenoid concentration by reflection spectroscopy. The pattern of the light reflected from the skin is mainly based on the presence of chromophores in the skin which contain carotenoids that absorb light at certain wavelengths. Taking into consideration the absorption spectrum of carotenoids, which is located in the blue-green range of the optical spectrum, the blue LED-emitted bright spectrum in the range between 440 nm and 490 nm was used as a source of excitation. The small dip in the reflected spectrum is based on the absorption by dermal carotenoids. The dermal carotenoid concentrations are determined by calculating the difference in intensity between the emitted and reflected spectra, and are expressed in arbitrary units. Prior to its application on bovine udder skin, the system was calibrated using Resonance Raman Spectroscopy [17]. A strong correlation was found (R = 0.81). Stability of the measurements was determined by the standard deviation of the measured values, which normally do not exceed 10% [15].

### Measurement protocol

**Figure 1:**
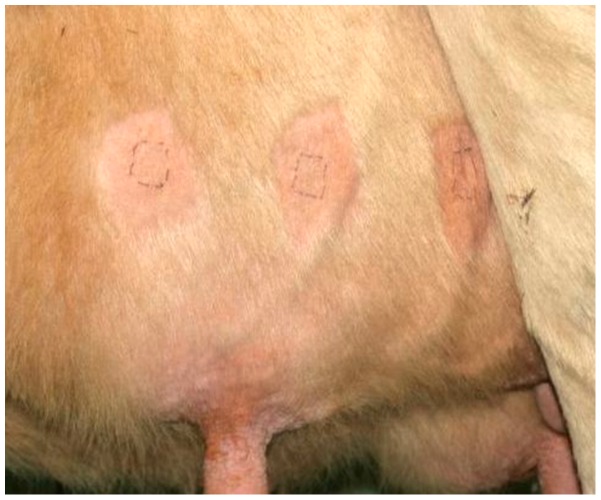
Measuring sites on the udder skin of a cow.

Measurements of dermal carotenoid concentrations took place once weekly over a period of one month as follows: The measurements were performed on the left body side on three pre-determined sites on the surface of the udder skin. The different sites were located 10 cm proximal to the basis of the teat. Two sites were located perpendicularly above the teats, while the third site was located halfway between these points. Prior to the measurements, the udder skin was carefully shaved without injuring the stratum corneum, using a single-use shaver, and the homogeneously pigmented skin sites of approx. 0.8 cm^2^ were marked (Figure 1). The MSS was placed on marked sites of the udder skin. Each site was subjected to three subsequent measurements. Measurements were performed in triplicate on each marked site. To this end, the MSS was removed from the skin between measurements and then positioned on the same skin area again. The time interval between the measurements was only a few seconds. This procedure has previously been described in detail by Klein et al. [16]. The median and the standard deviation were calculated from the triploid measurements at three different sites in close proximity.

### Serial determinations of the dermal carotenoid concentrations in six healthy cows (Trial 1)

In a first trial, six healthy non-lactating female cows belonging to the breeds German Holstein (n = 4), Uckermaerker (n = 1) and Simmentaler (n = 1) aged between two and 15 years were investigated. All animals were kept in tied stalls on straw bedding under the same environmental conditions, and received the same diet consisting of hay and grass silage ad libitum. In addition, each animal received 0.5 kg of Hendrix Illesch concentrate per day. None of the animals had calved in the six preceding months, was gravid or received any medical treatment. The carotenoid concentration was determined weekly over a period of four weeks.

### Course of dermal carotenoid levels following surgical treatment of abomasal displacement (Trial 2)

In a second trial, the dermal carotenoid levels were determined in 23 German Holstein dairy cows aged two to nine years (mean 4.37 years) that had been submitted to the Clinic for Ruminants in the period from January to April 2011 by local veterinarians due to abomasal displacement (DA). The animals originated from eight different dairy farms in the Brandenburg region and underwent surgery for DA either by endoscopical abomasopexy (Janowitz) or by celiotomy in the right flank followed by omentopexy (method according to Dirksen, Hanover, Germany). The first measurement took place within 12 hours after surgical intervention and was followed by repeated measurements during hospitalization. The last measurement was performed after dismissal at the farm of origin (between 20 and 60 days after surgery). In cases with a fatal outcome, the last measurement prior to euthanasia or death was considered for evaluation.

### Statistical Analysis

The software program SPSS 18.00 for Windows was used for the statistical analysis, with p<0.05 being considered statistically significant and p<0.001 being considered statistically highly significant. As part of the data was not normally distributed, the median was used for representation. The calculation was performed using non-parametric tests. While more than two dependent samples were subjected to Friedman's test, the Wilcoxon test was applied to compare two dependent samples.

### Ethics Statement

In the present study, all measurements were performed non-invasively according to §7 of the German Animal Welfare Act, which clarifies the necessity of ethical approval in the case of interventions or treatments involving pain, suffering or damage to the animals. Taking this definition into account, no application for ethical approval of animal experiments was filed, because it had been very clear already prior to commencement of the measurements that the animals would not be exposed to any pain, suffering or damage.

Both animal care and experimental procedures were approved and conducted under established standard of the Clinic for Ruminants and Swine, Faculty of Veterinary Medicine, Freie Universität Berlin and Charité - Universitätsmedizin Berlin, Germany.

## Results

### Repeated measurements on healthy cattle (Trial 1)

**Figure 2:**
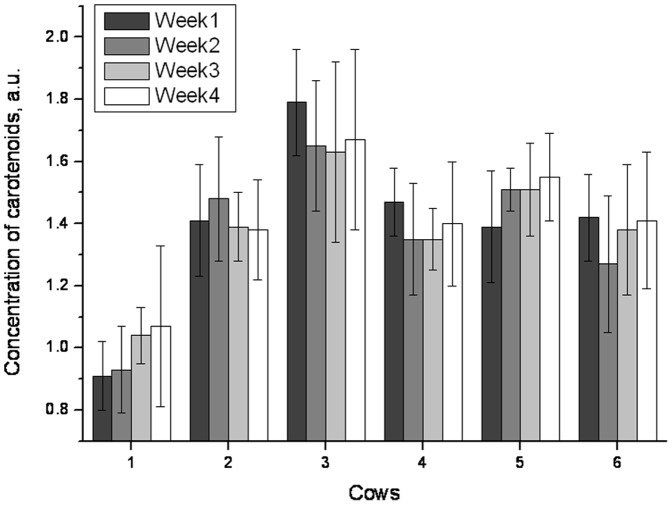
Course of dermal carotenoid concentrations expressed in arbitrary units in six healthy cows measured weekly over a period of one month.

The median of dermal carotenoid concentrations, as determined by reflection spectroscopy applied to three adjacent sites of the udder skin of six healthy cattle, ranged from 0.91 to 1.79 as expressed in arbitrary units (Figure 2). Significant differences were observed between individual animals kept under the same conditions. Serial measurements performed on single animals at weekly intervals over a period of one month delivered results that ranged from 0.99 to 1.66 as expressed in arbitrary units. No significant differences (p = 0.44) were observed when the results of repeated measurements in individual animals with time were compared (Figure 2 and Table 1). Mean deviations determined for individual cows within the time frame of one month amounted to ≤18%.

**Table 1:** Median and standard deviation (SD) of the nine individual values of the dermal carotenoid concentration as expressed in arbitrary units over a period of one month.

	week 1		week 2		week 3		week 4	
	median	SD	median	SD	median	SD	median	SD
cow 1	0,91	0.11	0.93	0.14	1.04	0.09	1.07	0.26
cow 2	1.41	0.18	1.48	0.2	1.39	0.11	1.38	0.16
cow 3	1.79	0.17	1.65	0.21	1.63	0.29	1.67	0.29
cow 4	1.47	0.11	1.35	0.18	1.35	0.1	1.4	0.2
cow 5	1.39	0.18	1.51	0.07	1.51	0.15	1.55	0.14
cow 6	1.42	0.14	1.27	0.22	1.38	0.21	1.41	0.22
Mean values	1.40	0.28	1.37	0.25	1.38	0.20	1.41	0.20

### Results of repeated determinations of dermal carotenoid concentrations in cows following surgical treatment of left abomasal displacement

**Figure 3:**
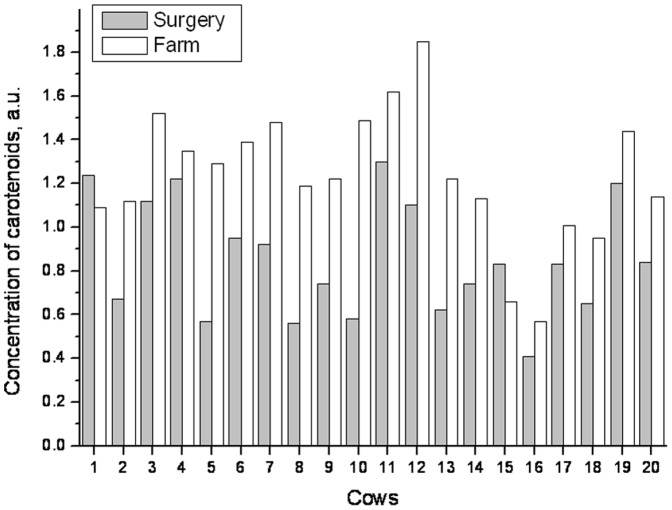
Course of the dermal carotenoid concentrations in 20 dairy cows treated surgically for abomasal displacement. Measurements were taken by reflection spectroscopy within 12 hours following surgery and at the farm of origin.

**Figure 4:**
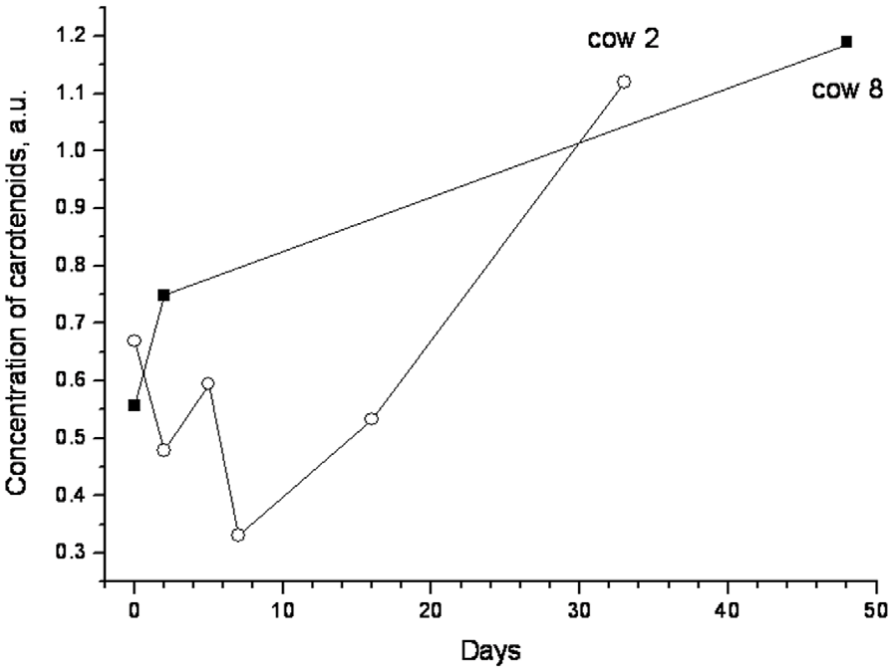
Course of recovery of cutaneous carotenoid concentrations in two cows from the day of surgery to the last measurement at the farm of origin. Cow 2 was discharged from the clinic two days post operationem due to the favourable development of its condition, while cow 8 stayed in the clinic until day 16 post operationem due to insufficient progress of its condition.

The results obtained from 23 cows which underwent surgery due to left displaced abomasum ranged from 0.41 to 1.30 arbitrary units in the perioperative phase (<12 hours post operationem). Six cows underwent surgery for DA by endoscopic abomasopexy (Janowitz) and 17 cows by celiotomy in the right flank followed by omentopexy (method according to Dirksen, Hanover, Germany). 3 cows (number 21, 22 and 23 in the Table 2) died or were euthanized, respectively, on day 3, 14 and 15 post operationem, they were measured at the farm of origin before their death. 18 of the 20 animals that recovered from left DA showed an increase in dermal carotenoid concentrations compared to the results of initial measurements, when examined after their dismissal on the farm of origin between day 20 and 60 post operationem (Table 2). Decreases were observed in two of the 20 cows (Figure 3). The mean increase in the 20 recovered cows was 53±44% compared to the initial value. Figure 4 illustrates the increase in the dermal carotenoid concentrations in two cows post operationem. The course of the dermal carotenoid concentrations was not consistent, but depended on the reconvalescence of the individual cow. Cow 8 was discharged from the clinic two days post operationem due to the favourable development of its condition. Its values kept increasing. Cow 2 stayed in the clinic until day 16 post operationem due to insufficient progress of its condition. Its dermal carotenoid concentration increased with considerable delay (Figure 4).

**Table 2:** Median and standard deviation (SD) of the dermal carotenoid concentrations in all 23 cows during their convalescence from DA involved in the study in the perioperative phase and at the farm of origin.

cow	day of surgery		at farm of origin	
	median	SD	median	SD
1	1.24	0.24	1.09	0.11
2	0.67	0.19	1.12	0.02
3	1.12	0.13	1.52	0.17
4	1.22	0.07	1.35	0.02
5	0.57	0.08	1.29	0.1
6	0.95	0.05	1.39	0.05
7	0.92	0.09	1.48	0.17
8	0.56	0.14	1.19	0.1
9	0.74	0.12	1.22	0.33
10	0.58	0.07	1.49	0.08
11	1.3	0.11	1.62	0.11
12	1.1	0.26	1.85	0.26
13	0.62	0.09	1.22	0.1
14	0.74	0.39	1.13	0.24
15	0.83	0.06	0.66	0.11
16	0.41	0.04	0.57	0.08
17	0.83	0.03	1.01	0.13
18	0.65	0.08	0.95	0.02
19	1.2	0.05	1.44	0.05
20	0.84	0.24	1,14	0.1
21	0.55	0.07	0.39	0.05
22	0.67	0.1	0.52	0.09
23	0.79	0.05	0.47	0.18
mean values	0.83	0.12	1.14	0.12

Cows 21–23 died after the surgery and were measured before the death at farm of origin.

20 days post operationem, the average median dermal carotenoid concentration of the 20 cows that had recovered exhibited a difference which was statistically highly significant (p<0.001) compared to the initial value.

**Figure 5:**
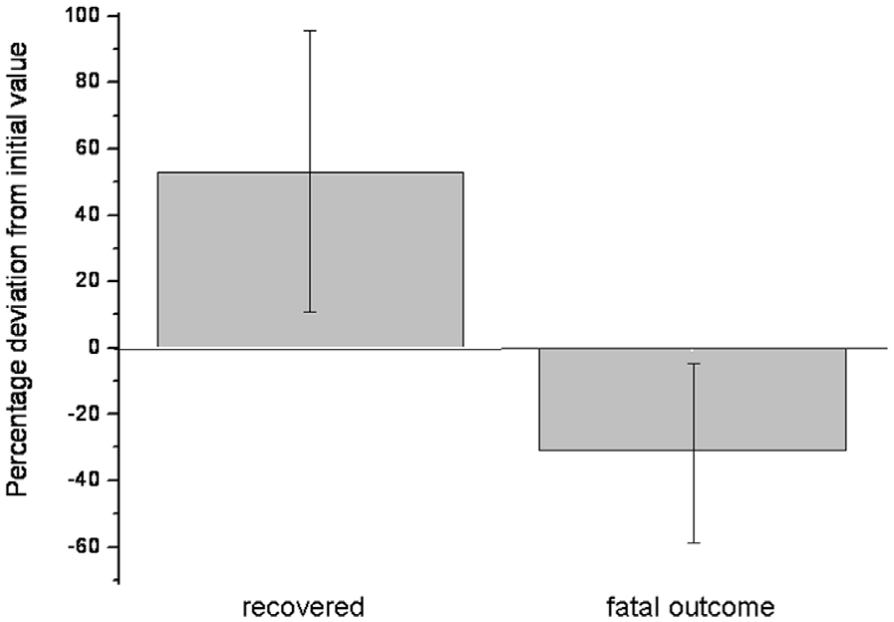
Percentage of increase and decrease in the cutaneous carotenoid concentration from the initial measurement to the final measurement at the farm of origin of the 20 recovered cows and to the last measurement prior to the death of 3 cows, respectively.

In contrast the levels decreased (mean 31±27%) in the three cattle in which the outcome was fatal (Figure 5).

## Discussion

### Repeated measurements in healthy cattle (Trial 1)

Measurements of the dermal carotenoid concentrations in six healthy cows kept under the same conditions rendered substantial interindividual differences. The latter findings are in accordance with earlier observations in healthy dairy cows that were examined at their farm of origin and that were kept under the same conditions and received the same diet [16]. When measuring isolated perfused cow udders ex vivo using reflection spectroscopy, Niedorf et al. also established a strong heterogeneity of the samples in terms of the β-carotene concentration in the skin [18]. Large interindividual differences regarding the β-carotene concentrations in the blood were also detected by both Lotthammer et al. and Noziere et al. [19], [20], [21]. Moreover, the dermal carotenoid concentrations in human volunteers covered a wide range of levels [22], [23], [24]. These findings were related to differences in the eating habits, lifestyles and, possibly, different stress conditions [1], [25], [26].

Repeated measurements in the same animal and the same skin area in weekly intervals revealed deviations of ≤18% which were not significant. Also the measurements taken within four days yielded no significant differences [16].

The udder skin was chosen as it can be easily accessed in cows and the light-coloured hair coat is only sparse in this region compared to the rest of the body. Measurements in humans have shown that the carotenoid concentration also depends on the side of the body on which the measurement is conducted [27], [28]. Contrary to the measurements reported by Niedorf et al. on an isolated perfused cow udder [18], [29], the data measured in this study were not influenced by absorption spectra of other chromophores due to the design of MSS [15]. The thickness of the epidermis of cow udder skin does usually not exceed 200 µm [30]. Taking into consideration the penetration depth of blue light into the skin, which is approximately 150–200 µm, as well as a design of MSS, the influence of dermal chromophores, such as melanin, haemoglobin, bilirubin, etc., is negligible [15]. Comparing β-carotene concentrations in the blood with dermal carotenoid concentrations in the same animal delivered significant correlations between the results of the two techniques for cattle with a moderate to obese body condition (unpublished data).

Using reflection spectroscopic measurements, a dose-dependent distribution of β-carotene in the skin and a decrease of the cutaneous β-carotene concentrations under UV irradiation could be demonstrated in an isolated perfused cow udder model [31]. Experiments in which β-carotene was administered to the model showed a satiety phenomenon with increasing concentrations and individually strongly varying increases in the cutaneous β-carotene concentrations. None of the β-carotene concentrations applied led to a plateau [18]. In human skin, the exposition to UV and IR radiation also induces a carotenoid reduction [32], [33], [34]. The cows subjected to the study were measured between January and early May, so that the cutaneous carotenoid concentration was not significantly affected by the varying influence of UV or IR radiation at the farms of origin.

### Course of dermal carotenoid concentrations following surgical treatment of DA (Trial 2)

DA is a common disease occurring in post partum dairy cattle. For this reason, dairy cows with DA that underwent surgery at a large animal hospital were chosen as probands to exemplify a disease condition in order to follow the course of dermal carotenoid concentrations over time until complete recovery. Reflection spectroscopy as performed in the present study does not provide absolute values but expresses dermal carotenoid concentrations in arbitrary units. As in healthy animals, significant differences were observed in the dermal carotenoid concentrations of cows with DA. When evaluating repeated measurements in single cows over time, those cows that recovered from DA showed an increase in their dermal carotenoid concentrations. Most likely this increase is a consequence of an increased appetite and the uptake of carotenoids with the feed as well as reduced consumption of dermal carotenoids due to decreased oxidative stress with the body functions returning to normal following reposition and fixation of the abomasum.

Hummel et al. [35] observed a highly significant improvement of the feed intake within the first seven days after abomasal surgery. Also, the ruminal activity, the characteristics of the excrements and the abdominal wall tension were normal to a large extent. The milk yield was also highly significantly increased in this period. In cows with left displaced abomasums, Gorber et al. could not detect wound swellings in any of the cows from day 20 post operationem [36]. Consequently, the animal is supposed to have completely recovered at day 20 post operationem and beyond. Due to the low number of cases, the significance was not calculated for the three cows that died or were euthanized.

Darvin et al. [1] and Vierck et al. [37] could show that illness and extreme stress also reduce the concentration of dermal carotenoids in humans due to formation of free radicals and especially reactive oxygen species. Investigations undertaken by Sattler et al. confirm that the antioxidative status of cows with abomasal displacement, measured based on the activities of superoxide dismutase and glutathione peroxidase, is affected by oxidative stress, with the severity depending on the duration and intensity of such stress [38]. In addition, the TEAC (Trolox Equivalent Antioxidative Capacity) concentrations were lower in the diseased cows (hoof and claw diseases, mastitis, metritis, abomasal displacement) than in the animals of the control group, with the time lapse to the partus being considered for comparison [39].

Many other studies have shown that both the carotenoid concentration in the blood and the antioxidative status as a whole is lower in cattle in the course of various diseases and in the periparturient period [40], [41], [42], [43]. It is supposed, therefore, that besides the stress that entailed the decrease in the carotenoid antioxidants, the reduced feed intake was the main reason for the reduced cutaneous carotenoid concentrations. As the general condition of the cows improved, they took in more feed and simultaneously their dermal carotenoid concentrations increased.

Reestablishment of hepatic functions is crucial for the recovery of cows with DA and the return to normal feed intake [44], [45]. Precision farming is a recent development in agriculture that includes introduction of recent innovations on the farm that allow cheap and fast determinations of parameters related with health and welfare of farm animals. Among these are determination of ketone bodies and urea in milk at milking. Future studies have to show if this novel scanning technique might be suitable for serial determinations of the oxidative status in farm animals in order to detect disorders that could cause harm to the health status of the animals at an early phase.

## Conclusions

Different cows show differences in the concentration of dermal carotenoids even if they are kept under identical conditions.

Under constant conditions, no significant differences were observed in median dermal carotenoid concentrations as determined in single animals by serial measurements over a period of one month.

In cows in which the recovery followed after abomasal displacement surgery, the average median dermal carotenoid concentration increased to a statistically highly significant value (p<0.001) when compared to the concentrations at initial measurements. In cows with fatal outcome, a decline of 31±27% in the dermal carotenoid concentration was detected during hospitalization.

Further studies are needed to test whether the scanner system is suitable for use in the concept of precision farming.
