# Correction: Serial Non-Invasive Measurements of Dermal Carotenoid Concentrations in Dairy Cows following Recovery from Abomasal Displacement

**DOI:** 10.1371/journal.pone.0114807

**Published:** 2014-11-25

**Authors:** The PLOS ONE Staff

The first author’s name is spelled incorrectly. The correct name is: Julian Klein. The correct citation is: Klein J, Darvin ME, Müller KE, Lademann J (2012) Serial Non-Invasive Measurements of Dermal Carotenoid Concentrations in Dairy Cows following Recovery from Abomasal Displacement. PLoS ONE 7(10): e47706. doi:10.1371/journal.pone.0047706
